# Advances in the development of new biomarkers for Alzheimer’s disease

**DOI:** 10.1186/s40035-022-00296-z

**Published:** 2022-04-21

**Authors:** Timofey O. Klyucherev, Pawel Olszewski, Alena A. Shalimova, Vladimir N. Chubarev, Vadim V. Tarasov, Misty M. Attwood, Stina Syvänen, Helgi B. Schiöth

**Affiliations:** 1 Department of Neuroscience, Functional Pharmacology, University of Uppsala, Uppsala, Sweden; 2 Department of Pharmacology, Institute of Pharmacy, I. M. Sechenov First Moscow State Medical University, Moscow, Russia; 3 Institute of Translational Medicine and Biotechnology, I. M. Sechenov First Moscow State Medical University, Moscow, Russia; 4 Department of Public Health and Caring Sciences, Rudbeck Laboratory, Uppsala University, Uppsala, Sweden

**Keywords:** Alzheimer's disease, Amyloid beta peptides, Biomarkers, Blood, Cytokines, Inflammation, MicroRNA, Ocular biomarkers

## Abstract

Alzheimer's disease (AD) is a complex, heterogeneous, progressive disease and is the most common type of neurodegenerative dementia. The prevalence of AD is expected to increase as the population ages, placing an additional burden on national healthcare systems. There is a large need for new diagnostic tests that can detect AD at an early stage with high specificity at relatively low cost. The development of modern analytical diagnostic tools has made it possible to determine several biomarkers of AD with high specificity, including pathogenic proteins, markers of synaptic dysfunction, and markers of inflammation in the blood. There is a considerable potential in using microRNA (miRNA) as markers of AD, and diagnostic studies based on miRNA panels suggest that AD could potentially be determined with high accuracy for individual patients. Studies of the retina with improved methods of visualization of the fundus are also showing promising results for the potential diagnosis of the disease. This review focuses on the recent developments of blood, plasma, and ocular biomarkers for the diagnosis of AD.

## Introduction

Alzheimer's disease (AD) is the most common neurodegenerative cause of dementia. Neurodegeneration (including atrophy and/or loss of neurons) is associated with toxic amyloid-beta oligomers and protein aggregates, intra-neuronal neurofibrillary tangles consisting of hyperphosphorylated microtubule-associated protein Tau, regionally specific reduction of cerebral glucose metabolism, synaptic dysfunction, and mitochondrial dysfunction [1–3]. The development of AD goes through three stages: the pre-symptomatic stage, the prodromal stage of mild cognitive impairment (MCI), and the clinical form of AD [4, 5]. AD accounts for 50%–70% of cases of common neurodegenerative dementia. It is estimated that about 44 million people worldwide are living with dementia, and this number could triple by 2050 due to an aging population [6]. Healthcare spending to care for people with dementia is estimated at $305 billion in 2020 [7]. The cost of AD for the US economy currently exceeds the cost of cancer or cardiovascular disease [8].

New methods to treat AD are being developed with variable success. A recent review suggests that there are about 28 agents in phase 3, 74 in phase 2, and 30 in phase 1 clinical studies, but the failure rate due to lack of evidence of effectiveness is high [9, 10]. Currently, there are no agents in clinical practice that could curtail the development of the disease and only symptomatic treatment is available. One possible reason for the lack of effectiveness in trials is the advanced stage of the disease at the time of pharmacological intervention [11]. It is hypothesized that at a certain neuropathological threshold, treatment can no longer affect the development of the disease [12]. Therefore, there is an urgent need for biomarkers that can identify patients with MCI and early stages of AD to achieve a robust effect from disease-modifying therapies. Currently, AD can be identified in patients at the preclinical stage in vivo, for example, by the biological or molecular signature of the disease [13]. For instance, in patients with dominantly inherited AD, a change in the level of cerebrospinal fluid (CSF) amyloid beta (Aβ) was detected at 25 years and CSF-P-Tau at 10 years before the onset of symptoms [14]. Cumulative Tau and Aβ pathologies, followed by cellular dysfunction in brain, lead to neurodegeneration which occurs shortly before clinical manifestation of AD, i.e., the onset of cognitive impairment [15]. The precise determination of neurodegenerative changes is challenging, since such changes are observed in cognitively normal aging individuals [16]. Furthermore, Tau pathologies alone can trigger neurodegeneration and the progression of Tau pathologies correlates with the severity of the cognitive impairment [17]. While Aβ and Tau pathologies have a well-documented impact on brain physiology in AD, their appearance and accumulation are a consequence of early impairment of immune functions and arising neuroinflammation [18]. There is epidemiological evidence linking AD and previous history of infection or diabetes, suggesting that inflammation can be a factor initiating AD pathology [19]. Indeed, Aβ which is normally cleared by microglia can induce an activation phenotype of microglia, leading to chemokine release and local inflammation [15, 20]. The spread of inflammation in turn affects Aβ clearance, and increases Tau phosphorylation and subsequent neurodegeneration [21]. Recently, a research framework regarding the diagnostic criteria has been articulated by the National Institute on Aging–Alzheimer’s Association [22]. This framework is intended for observational and interventional research and considers AD in a biological rather than a syndromal context with the use of an A/T/N classification system for AD biomarkers. In this system, "A" represents the concentration of Aβ biomarkers, "T" refers to the level of Tau biomarkers, and "N" reflects the biomarkers of neurodegeneration. This system allows the classification of AD markers according to the pathological mechanism and determines their participation in the pathogenesis of AD [22]. It is noteworthy that while the ATN classification provides precise metrics for AD diagnosis, it could be strengthened by the inclusion of other biomarkers such as brain vascularity changes, Lewy body pathology markers and aforementioned neuroinflammation [23].

Identification of AD biomarkers is an increasingly relevant area of research and many different approaches are being explored. One avenue is the feasibility of tracking the development of AD before the onset of symptoms, using plasma-based markers such as Aβ, Tau, and neurofilament light polypeptide (NFL). Monitoring of this group of markers could provide additional tools in clinical practice for the early diagnosis of AD and for the tracking of the effectiveness of AD therapies with Aβ-targeting drugs [24]. The potential use of Aβ and Tau as well as other proteins as biomarkers of the disease and the disease progression has been reviewed recently [25]. In addition to pathological proteins associated with AD, other promising groups of markers associated with neurodegeneration, inflammation, and lipid metabolism are also reviewed in this article. One of the interesting points in this work is the panel of potential biomarkers proposed by the authors as the most accurate and specific tool for diagnosing AD [25]. In parallel with protein markers, cell-free miRNAs can also be used for the diagnostics or monitoring of AD. In a recent systematic review, a network of 250 miRNAs associated with AD was cross-validated in the literature, which revealed a group of 10 miRNAs that could diagnose the disease 20 years before the onset [26]. One of the important aspects of AD biomarker development is the invasiveness of the test. Current diagnostic methods based on positron emission tomography (PET) imaging and protein analysis in the CSF are highly invasive and relatively expensive. Therefore, large efforts are being made on the search for favourable and minimally invasive biomarkers of AD based on sources of blood, saliva, ocular fluids, and olfactory fluid [27].

The primary focus of this analysis is to summarize evidence on three groups of markers that have the potential for use in clinical practice due to high specificity and sensitivity for the diagnosis of AD, as well as minimal invasiveness. We focus on the role of these biomarkers in the development of AD and their relationship with various aspects of this heterogeneous disease. We also summarize the existing problems and challenges in the search for biomarkers of AD and elucidate recommendations that could facilitate the development of new diagnostic tools.

## General overview of the different types of biomarkers

**Fig. 1:**
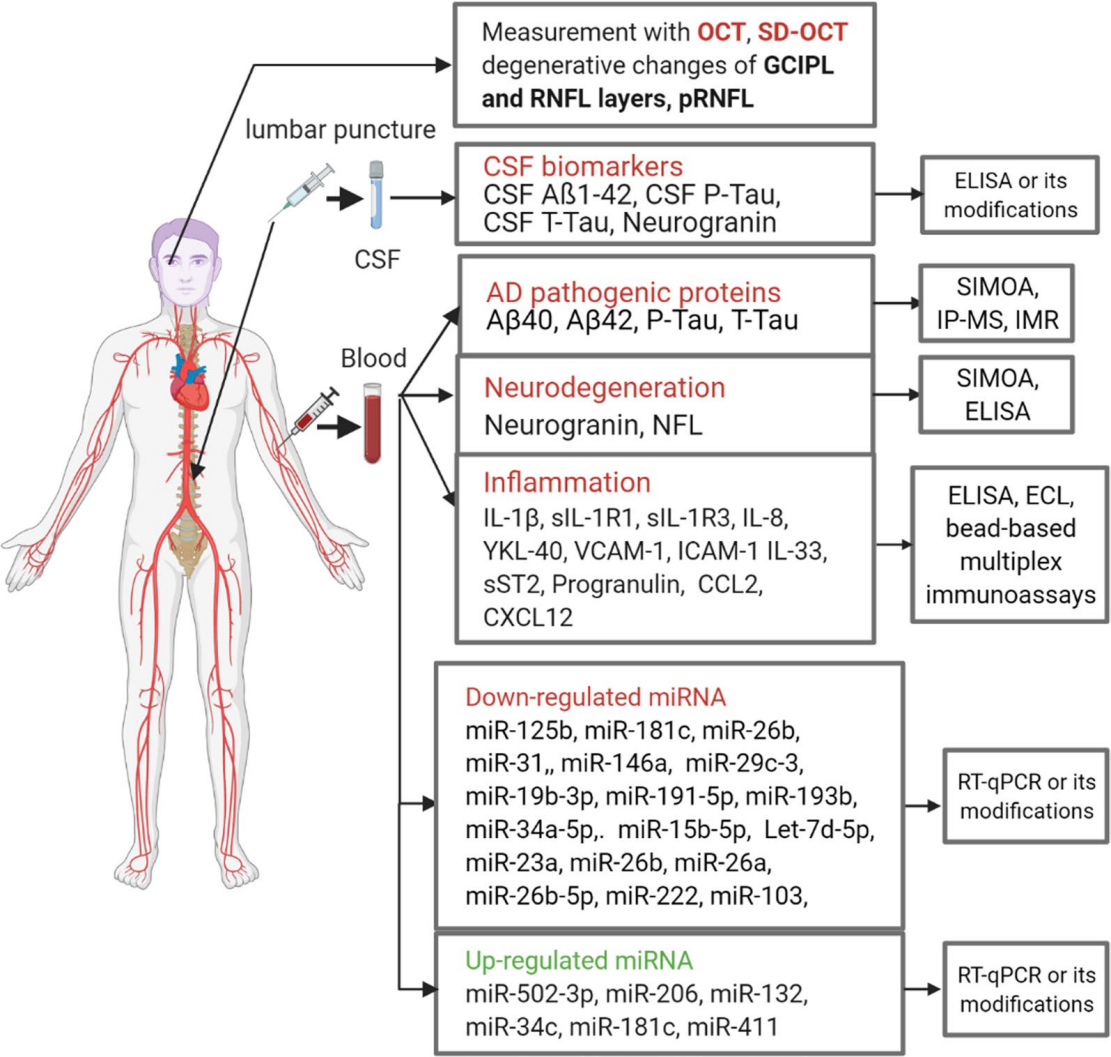
Classification of AD biomarkers. AD pathogenic proteins include the markers of the "amyloid theory", amyloid-beta (Aβ)_40_ and Aβ_42_, as well as markers of AD-related metabolic disorders, P-Tau (phosphorylated) and T-Tau (total). Biomarkers obtained by lumbar puncture are cerebrospinal fluid (CSF) biomarkers, CSF Aβ_1-42_, CSF P-Tau, CSF T-Tau, and Neurogranin. Neurodegeneration markers include neurogranin and neurofilament light (NFL). The markers of inflammation include IL-1β and two soluble receptors sIL-1R1 and sIL-1R3, IL-8, SDF-1, intercellular adhesion molecule 1 (ICAM1), vascular cell adhesion protein 1 (VCAM-1), progranulin, IL-33, and soluble interleukin 1 receptor-like (sST2). Many miRNAs are either up-regulated or down-regulated in studies on AD. The retina of the eye, as well as blood and plasma are being analyzed by a range of tools including single-molecular mass analysis (SIMOA), immunoprecipitation-mass spectrometry (IP-MS), immunomagnetic recovery (IMR), enzyme-linked immunosorbent assay (ELISA), and electrochemiluminescence immunoassays (ECL). Quantitative reverse transcription polymerase chain reaction (RT-qPCR) is used to identify miRNAs. The study of retinal degenerative changes, including ganglion cells and internal plexiform layers (GCIPL) and retinal nerve fiber layer (RNFL; p indicates peripapillary), is performed by optical coherence tomography (OCT) and spectral domain optical coherence tomography (SD-OCT)

The general classification of AD biomarkers considered in this review is illustrated in Fig. 1 and described below. We distinguish three major categories of biomarkers in AD: (1) diagnostic markers—PET imaging and CSF analysis for Aβ and Tau; (2) blood markers—protein and miRNA biomarkers analyzed in the whole blood, plasma or serum; and (3) ocular markers—the least invasive methods based on the identification of AD-associated changes in the retina. The present review is focused on blood and ocular biomarkers as novel approaches, while biomarkers used in clinical diagnostics have been reviewed extensively elsewhere [28, 29].

1. Diagnostic markers

Today, imaging methods, including structural magnetic resonance imaging (MRI) for visualization of brain atrophy, ^18^F-2-fluoro-2-deoxy-*D*-glucose ([^18^F]FDG) PET for measurement of brain metabolism, amyloid-PET for quantification of insoluble Aβ deposits (plaques), Tau-PET [^18^F] flortaucipir for quantification of pathogenic Tau and CSF biomarkers (CSF Aβ_42_ and Tau) are recognized as valid diagnostic tools [22, 30]. A large number of amyloid-PET studies report around 90% senisitivity and specificity to diagnose AD with only minor differences between the different available radioligands [31, 32]. Tau PET has also demonstrated a high selectivity to distinguish AD dementia from other neurodegenerative disorders and is superior in diagnostic accuracy compared to MRI markers [33]. Preclinical AD has been confirmed in asymptomatic patients at a risk of developing clinical AD, whose brain amyloidopathy revealed by PET may precede the onset of the prodromal stage or dementia stage by several years [34].

CSF Aβ_1-42_, hyperphosphorylated Tau peptide (P-Tau), and total Tau protein (T-Tau) have a high diagnostic accuracy exceeding 90%, especially when a combination of these CSF biomarkers is used for diagnosis [35]. In addition, CSF markers can reveal changes that precede PET abnormalities, which is relevant for early detection [36]. However, the use of PET and CSF sampling is limited due to the high cost and invasiveness, respectively. Currently, most patients who undergo amyloid-PET imaging do so as part of their participation in a clinical trial [37]. Some patients experience CSF sampling as painful since it requires a lumbar puncture, and it often takes weeks to get results due to the lack of laboratory facilities that perform fluid analysis. However, the cost of CSF analysis is lower than that of amyloid PET scanning [31]. The high cost of PET and the invasiveness of CSF sampling are major obstacles to their population screening use to detect potentially manageable pre-clinical AD [38]. Based on this, there is a need for modern, reliable, low-cost, selective, and less invasive methods for diagnosing AD and distinguishing AD from other neurodegenerative diseases, such as MCI, or mixed forms of dementia, such as vascular dementia, frontotemporal dementia and Lewy body dementia (LBD). It should be mentioned that the spread of co-occurring pathologies significantly complicates the diagnosis of AD, and detection of mixed pathologies is still difficult and might lead to misdiagnosis [39, 40]. It is possible to accurately separate AD from other neurodegenerative diseases only if biomarkers for non-AD-related diseases are developed in parallel to the development of AD biomarkers.

(2) Blood and fluid biomarkers

Blood-based and fluid biomarkers, especially measurement of Tau in plasma, are emerging. Blood-based biomarkers have an advantage of compatibility with primary health care use since the blood sampling can be easily performed and does not require complex training. The measurement of blood-based biomarkers could also be an ideal first step in a multi-step diagnostic process. Primary health care facilities could conduct screening to identify patients who may require an additional evaluation by specialists, including CSF diagnostic analysis, MRI, or amyloid PET diagnostics [41]. However, to achieve wide-spread use in primary healh care, it is necessary to reduce the excessive variability in plasma biomarkers. As another potential group of blood-based biomarkers, miRNAs have been identified in plasma, serum, and CSF [42] as markers of diseases, such as cardiovascular diseases, cancer, and neurodegenerative disorders [43]. A number of studies have shown that almost 50% of known miRNAs are expressed in the nervous system, and play an important role in regulating normal brain physiology, as well as in aging and mental illness [44]. In AD, miRNAs target key disease genes, showing either neurodegenerative or neuroprotective effects [45].

(3) Occular markers

Eye scans using high-resolution imaging technologies, such as optical coherence tomography (OCT) to diagnose AD at an early stage, are also gaining increasing interest. A number of studies in AD animal models have revealed degenerative changes in the retina [46–50]. Moreover, there is abundant evidence in the literature describing degenerative changes in the eyes of patients suffering from AD, which suggests a link between ocular pathology and the development of neurodegeneration [51–55]. Particularly interesting are those studies that demonstrated a correlation for retinal changes in patients before they showed symptoms of dementia compared to controls [56].

## Blood-based biomarkers of protein pathology

### Aβ in plasma

Aβ is the main component of the insoluble protein inclusions, plaques, found in AD brains. The protein exists in two different forms: pathogenic Aβ_42_, which is localized in diffuse amyloid aggregates, and Aβ_40_, which is localized in the core of mature plaques [57]. Numerous studies indicate that the plasma Aβ could be a cost-effective alternative to conventinal CSF-based markers for identification of AD [58]. However, the clinical implementation has been hampered by the inconsistency among results, as well as the insignificant changes of Aβ_42_ and Aβ_40_ levels in blood plasma in contrast to CSF Aβ in AD patients [59, 60]. This issue may be related to the Aβ epitope masking by its binding to plasma proteins, which is an analytical shortcoming of enzyme-linked immunosorbent assay (ELISA) or other standard immunoassays [61] that are commonly used to assess the levels of Aβ in plasma. This lack of association between Aβ in plasma and CSF might also be due to the Aβ expression by cells in peripheral tissues such as platelets [62], skin fibroblasts [63] or skeletal muscles [64], which contributes to the total Aβ plasma concentration [65].

Antoher challenge with the use of plasma Aβ as a biomarker is the much lower concentrations of Aβ_40_ and Aβ_42_ in plasma compared to CSF [59, 60]. However, the emergence of hypersensitive methods, such as single-molecular mass analysis (SIMOA) and immunoprecipitation-mass spectrometry (IP-MS), enables detection of minor changes in the Aβ plasma level in patients with AD. Blennow and Zetterberg have demonstrated that the accurate quantitation of plasma Aβ_42_ level to sub-picograms per millimeter (limit of quantitation, 0.04 pg/ml) can be achieved with SIMOA [66]. In a previous study, this assay was used to quantitate Aβ_40_ and Aβ_42_ levels in blood plasma and CSF of 274 controls, 174 patients with subjective cognitive decline (SCD), 214 MCI and 57 AD (Swedish BioFINDER cohort) [59]. Results revealed reduced levels of Aβ_42_ in blood plasma of AD patients compared to the control group (13.2 ± 7.3 pg/ml in AD vs 19.6 ± 5.2 pg/ml in control). In patients with pathological CSF signature, the level of Aβ_42_ in blood plasma decreased gradually in SCD (17.4 ± 5.6 pg/ml), MCI (17.6 ± 4.9 pg/ml) and AD (12.9 ± 7.1 pg/ml) compared to controls (18.3 ± 4.2 pg/ml). However, only the AD patients with pathological CSF signature showed significant difference in plasma Aβ_42_ from control, suggesting a limited potential of this biomarker for distinguishing pre-clinical AD with CSF pathologies. The concentrations of Aβ_40_ for SCD (238.7 ± 105.5 pg/ml) and MCI (284.3 ± 72.8 pg/ml) were not different from the control group (274.6 ± 70.9 pg/ml) [59]. A recent application of the IP-MS method showed that the Aβ_42_/Aβ_40_ ratio was 14% lower in the amyloid PET-positive group (Aβ_42_ 37.13 pg/ml, Aβ_40_ 288.0 pg/ml) compared to the age-mathced controls [67]. Interestingly, the plasma composite biomarker (normalized scores for APP_669–711_/Aβ_142_ and Aβ_140_/Aβ_142_) demonstrated a strong relationship (and 80.4% accuracy) between plasma and CSF levels among patients with AD, showing a comparable performance to CSF Aβ_42_ in determining Aβ burden in the brain [60]. Thus, although sensitive anaylsis methods are emerging, the use of plasma Aβ as a biomarker is most likely to become a useful diagnostic strategy mainly in combination with other diagnostic means.

### Plasma Tau

The major physiological role of Tau is to stabilize microtubules in neuronal axons [68]. In AD, neuroaxial degeneration leads to increased Tau release from neurons. In addition, Tau undergoes truncation and phosphorylation, which leads to its aggregation in the neurofibrillary tangles of the proximal axoplasm [69]. Abnormally phosphorylated and truncated Tau protein is the main component of neurofibrillary tangles in AD and other tauopathies [70]. The CSF total Tau might be a non-specific marker, as it is also elevated after traumatic brain injury and acute stroke as a marker of neuronal death [71, 72]. However, increased levels of phosphorylated Tau at specific sites, such as P-Tau181, P-Tau217, and P-Tau231 in CSF and blood are regarded as AD-specific biomarkers [23, 73, 74]. The specificity of this biomarker is supported by the fact that increased levels of P-Tau in CSF reflect the formation of neurofibrillary tangles in the brain [75].

In line with plasma Aβ, one challenge for the development of blood-based Tau is the relatively low concentration of Tau in the blood, which is significantly lower than that in the CSF. The CSF level of Tau is about 2–300 pg/ml, while the plasma concentration is approximately 100 fold lower, about 5 pg/ml [76]. Application of SIMOA and Meso-Scale methods to detect plasma P-Tau181 and P-Tau217 in large patient cohorts has confirmed their increase in AD dementia, although with a significant overlap with the control groups [74, 77, 78]. A recent study on the Alzheimer's Disease Neuroimaging Initiative (ADNI) and BIOFINDER groups showed that the plasma P-Tau, as well as other biomarkers, demonstrates different concentrations in patients converting to AD, in comparison with individuals who do not progress to dementia (BioFINDER: P-Tau217 0.40 ± 0.25 vs 0.17 ± 0.14 pg/ml, P-Tau181 4.20 ± 2.22 vs 2.26 ± 4.54 pg/ml; ADNI: P-Tau181 24.4 ± 10.8 vs 15.8 ± 11.4 pg/ml for converting vs non-converting) [79]. Nevertheless, in all cases the levels of pathological Tau phosphorylated at threonines (T) in positions 181 and 217 were significantly higher (*P* < 0.001) in AD than in control groups.

In addition, longitudinal data from the ADNI and BioFinder groups have also demonstrated a significant association between P-Tau and cognitive impairment, atrophy, and hypometabolism during follow-up. In the ADNI group, higher plasma Tau levels (P-Tau from 3.44 pg/ml to 8.89 pg/ml) predicted a rate of cognitive decline, an increase in brain atrophy measured with MRI, and a decrease in cortical glucose metabolism as shown by FDG-PET [80]. More recently, longitudinal analysis of both the ADNI and BioFinder data demonstrated that the P-Tau levels in subjects with SCD or MCI predicted their conversion to AD with high accuracy [79]. The prediction was improved if the plasma P-Tau level was combined with a brief cognitive test and apolipoprotein E (APOE) genotyping (where the *APOE* ε4 allele is a main risk factor for AD). The prediction was not improved by replacing these tests with CSF measurement of P-Tau, Aβ_40_ and Aβ_42_, indicating that a selection of appropriate non-invasive biomarkers may be sufficient for clinical prediction and diagnosis of AD in the future.

## Fluid biomarkers of synaptic degeneration

### Neurogranin

Neurogranin is a calmodulin-binding protein expressed in brain areas most affected by AD, such as the cortex and hippocampus, which can be used to reflect synaptic loss [81]. Neurogranin has a key influence on synaptic plasticity and increases synaptic strength by regulating calmodulin availability by Ca2þ-mediated activation of protein kinase C [82]. Neurogranin has been suggested as a CSF marker of AD neurodegeneration that could reflect synaptic degeneration. A number of cross-comparisons have revealed an increase in CSF neurogranin in patients with AD and MCI compared to healthy control groups [83, 84]. ELISA assay using newly developed monoclonal antibodies for neurogranin has revealed high levels of neurogranin in CSF, which are capable of predicting prodromal AD and MCI at a concentration of 336 pg/ml (IQR 126–505) and 210 pg/ml (IQR 83–433), respectively [85]. The ADNI study confirmed high levels of CSF neurogranin in AD dementia and prodromal AD. A high level of CSF neurogranin at 382 pg/ml also correlates with the future rates of hippocampal trophism by MRI detection and metabolic contractions by FDG-PET [86]. At the same time, there is evidence that elevated levels of CSF neurogranin (175.5 ± 217.8 pg/ml vs 99.2 6 ± 102.9 pg/ml for Aβ+ and Aβ− MCI patients, respectively) indicate future cognitive decline in Aβ+ MCI patients [87]. These dynamic changes in CSF neurogranin concentrations may reflect different stages of AD [88]. It has been found recently that the CSF neurogranin is elevated exclusively in patients with AD but not in other neurodegenerative disorders, such as frontotemporal dementia, LBD, Parkinson's disease, progressive supranuclear palsy, or multiple system atrophy. At the same time, a study examining plasma neuronal-derived exosomes showed that patients with AD and frontotemporal dementia displayed lower levels of plasma neuronal-derived exosomal neurogranin (232 ± 56.5 pg/ml and 1117 ± 227 pg/ml, respectively) compared to the control group (2208 ± 354 pg/ml) [89]. These findings suggest that the CSF neurogranin might be a specific marker for AD, but more studies are needed [90, 91].

### NFL

NFL is a native cytoskeletal protein that can be used as a plasma biomarker of axon damage [92]. In several studies, CSF NFL performed better than Aβ and Tau proteins in reflecting future cognitive decline and the clinical severity of AD and MCI [87, 93]. The NFL level in CSF has a good correlation with plasma NFL level, although the former is found in significantly higher concentrations [94, 95]. The baseline NFL concentration in blood is already distinguishing AD and MCI from controls (45.9 pg/ml for AD, 37.9 pg/ml for MCI and 32.1 pg/ml for controls), and an increase of NFL concentration is observed with AD progression, which correlates with the decline in cognitive functions [92]. The potential of plasma NFL to serve as an AD biomarker is corroborated by its ability to distinguish patients with genetic predispositions to AD. In a recent study, the annual change rate of NFL allowed for separation of carriers of the *APOE* ε4 allele, a risk factor for AD, from non-carriers 16 years before the expected onset of symptoms [94], rendering NFL as a possible biomarker for preclinical AD. However, since NFL is a component of the cytoskeleton of neuronal cells, its level may increase in many neurodegenerative diseases including Creutzfeld-Jakob disease, amyotrophic lateral sclerosis, frontotemporal dementia, HIV-associated dementia, and others [96]. In a cohort of ADNI patients, the plasma NFL level correlated with brain atrophy only in symptomatic patients, that is, MCI and AD groups. In contrast, CSF NFL concentration showed a negative correlation with the cortical thickness also in controls with and without pathological Aβ. In addition, neurogranin showed a greater association with and specificity for AD pathology compared to the plasma NFL. Thus, NFL may not be a specific marker for AD in contrast to neurogranin [95]. Also, not all studies of the NFL marker provided consistent results. For example, in a longitudinal study, a correlation was observed between elevated plasma NFL levels and neuropsychological scores; the baseline plasma NFL level was higher in AD dementia (26.49 ng/ml) than in MCI (17.77 ng/ml) (standardized mean difference = 0.55, 95% CI 0.37–0.73) and normal cognition (15.33 ng/ml)(standardized mean difference = 0.68, 95% CI 0.49–0.88), according to the Clinical Dementia Rating scores (OR = 1.94, 95% CI 1.35–2.79). Longitudinally, NFL did not predict diagnostic conversion, but the plasma NFL level significantly correlated with worse performance in all 10 neuropsychological tests and measures of verbal fluency and episodic memory. It should be noted, however, that during the follow-up, only 3 of these tests remained statistically significantly associated with the baseline plasma NFL [97]. Thus, the plasma NFL could be used in clinical practice as an auxiliary biomarker that confirms the presence of neurodegeneration. The aforementioned research results generally support that the plasma NFL can be used to monitor neurodegeneration and predict the severity of the disease.

## Biomarkers of neuroinflammation

One of the important factors in AD disease progression is the chronic neuroinflammation, and microglia play an important role in this process. A number of genes are expressed during neuroinflammation, including ATP-binding cassette sub-family A member 7 (*ABCA7*), sialic acid binding Ig-like lectin 3 (*CD33*), complement receptor type 1 (*CR1*), ephrin type-A receptor 1 (*EPHA1*), membrane-spanning 4-domains (*MS4*), and triggering receptor expressed on myeloid cells 2 (*TREM2*) [98]. Elevated levels of pro-inflammatory molecules in the brain lead to increased neurological deficits and increase the permeability of the blood–brain barrier (BBB). Proteins and genes related to neuroinflammation are currently considered as targets for disease-modifying drugs [99], and thus, novel biomarkers related to neuroinflammation are also of interest. Interleukins (IL) are potential biomarkers investigated in many studies, as the concentrations of proinflammatory cytokines including IL-1α, IL-1β, and IL-6 are altered in AD [100, 101]. Chemokines also play an important role in inflammation. For example, patients with AD show increased monocyte chemoattractant protein CCL2 in serum and CSF compared to the control group [102]. A similar pattern has been observed for interferon gamma-induced protein 10, IL-8 [103], and stromal cell-derived factor-1 [104]. However, the changes obtained may not be directly associated with AD, and it is possible that these changes can be explained by aging or the influence of a systemic disease.

Progranulin, a growth factor enhancing the growth of neurons and their survival, has been shown as a possible marker for early prediction among patients [105, 106]. This growth factor is expressed in neurons and microglia and participates in neuroinflammatory modulation, reducing microgliosis and astrogliosis [107]. The CSF level of progranulin (1082 pg/ml) increases as early as 10 years before the clinical presentation of the disease in patients with familial AD and late-onset sporadic AD [108]. In another study, researchers found increased expression of *GRN* (which encodes progranulin) in the blood of AD and MCI patients in three out four tested cohorts; however, these results did not correlate with plasma granulin concentration which did not differ among groups [109]. These results might be explained by the fact that the current ELISA methodology does not allow detecting changes in progranulin due to the complex post-translational changes occurring with this protein [109]. YKL-40, known as chitinase-3-like protein 1 (encoded by the *CHI3L1* gene), is a chitin-binding lectin belonging to the glycosylhydrolase family 18 [110]. The expression of YKL-40 increases in astrocytes under conditions of neuroinflammation [111]. In a longitudinal study on cognitively healthy people at risk of developing AD, the plasma concentration of YKL-40 was negatively correlated with the deposition of Aβ in the brain and positively correlated with the results of sensitive Free and Cued Selective Reminding Test (FCSRT) [112]. During the study, the average concentration of YKL-40 measured by ELISA increased from baseline 10.83 ± 0.62 pg/ml to 11.03 ± 0.56 pg/ml after 36 months of observation and a positive associatin between age and YKL-40 was observed. While these results are promising regarding the use of YKL-40 as an AD biomarker, this study had some limitations and further longitudinal studies on larger cohorts (small effect size of the current study) with more detailed clinical (Aβ–PET diagnostics) and genetic (e.g. *TREM2* variants) tests are required [112].

Intercellular adhesion molecule 1 (ICAM-1) and vascular cell adhesion molecule-1 (VCAM-1) are cell-surface glycoproteins on endothelial cells and immune cells, mediating the adhesion of leukocytes to endothelial cells and the transport of leukocytes to the brain, and they reperesent another group of interesting markers of inflammation in AD [113]. Elevated levels of VCAM-1 and ICAM-1 in the elderly correlate with increased levels of C-reactive protein (CRP) and are associated with microvascular endothelium-dependent vasodilation [114]. In one of the studies, the plasma levels of VCAM-1 measured by ELISA in patients with AD (708 ng/ml), as well as in patients with vascular dementia (728 ng/ml), were increased by 1.3 times compared to non-demented control patients (562 ng/ml) [115]. In patients with AD, an increase in the level of serum-soluble ICAM-1 was observed compared with non-inflammatory neurological diseases (NINDs) and non-diseased controls [116]. The CSF level of ICAM-1 was increased in patients with AD at the early, preclinical, and MCI stages, and correlated with the severity of cognitive decline [117]. IL-33 and the soluble form of its receptor ST2 (sST2) could be other biomarkers of inflammation in AD. IL-33 is commonly associated with inflammation; however, in AD it plays a protective role through stimulation of microglia and subsequent reduction of Aβ plaques in mouse models [118]. IL-33 is downregulated in the brain tissues of MCI and AD patients; however, its concentration in plasma is higher in MCI and AD compared to healthy controls [119]. MCI and AD patients with positive expression of IL-33 in serum had better performance in cognitive tests in a 1-year follow up, which further underscores the beneficial role of IL-33 [120]. Since higher levels of IL-33 in plasma of MCI and AD patients are associated with better cognitive function, it is surprising why this cytokine is elevated in AD and MCI at all. A possible explanation of this pehonomenon is suggested by recent studies which showed that AD patients have elevated level of sST2, which ameliorates physiological effects of IL-33 and can contribute to the decline of cognitive function in the course of AD [119, 120]. Therefore, in future studies measurement of combined IL-33 and sST2 in the context of AD is needed.

Inflammatory markers are potential supplements to the AD marker panel. Most of these inflammatory biomarkers can be isolated from blood or its derivatives, whose concentrations are typically measured by ELISA (or other immunoassays, such as electrochemiluminescence immunoassay, including Mesoscale Discovery immunoassay or V-PLEX [117]), thus offering relatively easy scalability in clinical adoption. Further research is needed on a broad cohort of patients to clarify their specificity for AD and whether they can be used to predict cognitive decline and track the effectiveness of AD therapies aimed at reducing neuroinflammation.

## Other protein biomarkers of AD

Epidermal growth factor (EGF) signaling can affect the process of neurogenesis in adults [121]. In a multiplex immunoassay of the ADNI cohort using a Luminex xMAP platform and a study of presymptomatic to late-stage AD patients using arrayed sandwich ELISA, patients with AD were shown to have a decreased EGF level compared to the NC group [122, 123]. Another interesting biomarker of AD is the pancreatic polypeptide, which is elevated in plasma of patients with AD and MCI. An increased level of pancreatic polypeptide may reflect the loss of neurons, regardless of the etiology, and also demonstrate a violation of transport through the BBB [124]. Identification of novel, potential protein biomarkers is facilitated by the emergence of modern methods, allowing for large-scale proteomic studies of AD biomarkers to yield promising results. An example of such method is the proximity extension assay (PEA), which is a modified immunoassay utilizing two antibodies per protein in the aim to increase the specificity and DNA-tags which are amplified and quantified after extension [125]. Through the specific interaction of antibodies with their epitepoes, this method overcomes the problem of high abundance of albumin which consists of approximately 55% of plasma protein [126]. In one of the recent studies utilizing PEA, 270 proteins in the plasma and CSF were examined for several cohorts of patients with early-onset AD [127]. Among the proteins identified as novel biomarkers were proteins associated with innate and acquired immunity (YKL-40, chitinase 1), junctional adhesion molecule B, matrix metallopeptidase 10, tumor necrosis factor-related activation-induced cytokine (TRANCE/RANKL), tumor necrosis factor-related apoptosis-inducing ligand, cell adhesion and differentiation (activated leukocyte cell adhesion molecule, ALCAM), repulsive guidance molecule BMP co-receptor b, axin-1 (AXIN1), and eukaryotic translation initiation factor 4E-binding protein 1. Another study utilized PEA to analyze 429 plasma proteins, among which a panel of 19 proteins was proposed to have high diagnostic accuracy for AD [128]. The proposed panel can determine the specific stage of AD, which makes it particularly useful for early diagnosis and progression monitoring. For example, three proteins NEL-like protein 1, human kallikrein 14, and centrin-2 were detected at an early stage of AD and changed throughout the progression of the disease, whereas tyrosine-protein kinase Lyn, protein kinase C theta, and the leukemia inhibitory factor receptor showed changes in the initial and intermediate stages of the disease [128]. An interesting area of research is the compilation of genomic atlas of the proteome involved in neurodegenerative diseases. Recently, a genomic atlas of AD-related protein levels has been created in CSF, plasma, and brain tissues. In that study, 1305 proteins were studied in patients with AD, among which 274, 127, and 32 loci of quantitative protein signs in the CSF, plasma, and brain were identified [129]. A summary of the fluid biomarkers of AD and their reported changes in AD are shown in Table 1.

**Table 1:** Summary of fluid biomarkers and their changes in AD

Biomarker	Study group	Sample	Remark	References
Aβ_1-42_/Aβ_1-40_	AD *n* = 57 MCI *n* = 214 SCD *n* = 174 NC *n* = 274	Plasma CSF	AD Aβ_42_ and Aβ_40_ AD ↓ vs NC	[59]
APP_669–711_/ Aβ_1-42_ Aβ_1-40_/Aβ_1-42_	Cohort NCGG AD *n* = 29 MCI *n* = 30 NC *n* = 62 Cohort AIBL AD *n* = 29 MCI *n* = 67 NC *n* = 156	Plasma CSF	AD plasma composite biomarker and other Aβ bimarkers (including Aβ_42_) showed significant correlations with Aβ-PET	[60]
P-Tau	AD *n* = 26	CSF	AD CSF P-Tau showed a positive correlation with counts of neurofibrillary tangles (NFT) and neuritic plaques (NP)	[75]
T-Tau	AD *n* = 54, MCI *n* = 75 NC *n* = 25	Plasma CSF	T-Tau ↑ in AD	[130]
Aβ_1-40_ Aβ_1-42_ T-Tau	AD/MCI *n* = 25 NC *n* = 20	Plasma	Aβ_42_↑ in AD Aβ_42_/Aβ_40_↑ in AD T-Tau↑ in AD	[131]
T-Tau P-Tau	AD *n* = 28, NC *n* = 23	CSF	T-tau↑ in AD	[132]
Aβ_1-42_ T-Tau	Cohort ADNI: AD *n* = 179 MCI *n* = 195 NC *n* = 189 Cohort BioFINDER: AD *n* = 61 MCI *n* = 212 SCD *n* = 174 NC *n* = 274	Plasma CSF	AD Plasma Tau ↑ vs NC and MCI (ADNI cohort) CSF Aβ_42_ negatively correlated with Plasma Tau (ADNI cohort) AD plasma Tau > Aβ− NC, Aβ+ NC, Aβ− MCI, Aβ+ MCI (ADNI cohort) Aβ+ MCI plasma Tau > Aβ− MCI (ADNI cohort) CSF Aβ_42_ positively correlated with CSF T-Tau and P-Tau (BioFINDER)	[80]
NFL, T-Tau	AD *n* = 156 MCI *n* = 185 NC *n* = 279	Plasma	Plasma NFL AD↑ vs MCI and NC	[97]
P-Tau, T-Tau	AD *n* = 40 MCI *n* = 57 NC *n* = 172	Plasma	T-Tau↑ in AD and MCI P-Tau ↑ in AD	[78]
Neurogranin	AD *n* = 65 MCI *n* = 61 NC *n* = 37	CSF	Neurogranin ↑ in AD and MCI baseline Neurogranin ↑ in AD and MCI follow-up	[83]
Neurogranin YKL-40	AD *n* = 74 DLB/PDD *n* = 47 VaD *n* = 34 FTD *n* = 33 NC *n* = 53	CSF	Neurogranin ↑ in AD and MCI vs NC YKL-_40_ ↑ in AD vs NC	[84]
Neurogranin NFL YKL-40 T-tau	AD *n* = 180 MCI *n* = 450 NC *n* = 140	CSF	Neurogranin ↑ in Aβ+ vs Aβ− NFL ↑ in AD and MCI vs NC YKL-_40_ ↑ in AD and MCI vs NC T-tau ↑ in AD and MCI vs NC	[87]
Neurogranin	AD *n* = 100 MCI *n* = 40 NC *n* = 80	CSF	Neurogranin ↑ in AD and MCI	[85]
Neurogranin	AD *n* = 95 MCI *n* = 173 NC *n* = 110	CSF	Neurogranin ↑ in AD and MCI	[86]
Neurogranin	AD *n* = 100 genetic AD *n* = 2 bvFTD *n* = 20 svFTD *n* = 21 DLB *n* = 13 PD *n* = 31 PSP *n* = 46 MSA *n* = 29 NC *n* = 50	CSF	Neurogranin ↑ in AD and genetic AD	[90]
Neurogranin	AD *n* = 397 MCI *n* = 114 NC *n* = 75	CSF	Neurogranin ↑ in AD	[91]
Neurogranin	Cross-sectional study AD *n* = 12, FTD *n* = 16, NC *n* = 28 Longitudinal study AD *n* = 9 FTD *n* = 10 NC *n* = 19	Plasma	Exosomal neurogranin ↓ in AD	[89]
NFL	Mut AD *n* = 243 NC *n* = 162	CSF Serum	NFL ↑ in AD in serum and CSF	[94]
IL-1β	AD *n* = 58 MCI *n* = 74 NC *n* = 31	Serum	IL-1β ↑ in AD and MCI	[101]
CCL2 (MCP-1)	AD *n* = 41 NC *n* = 31	serum	CCL2 ↑ in AD	[133]
CXCL12	AD* n* = 30 NC *n* = 30	Serum	CXCL12 ↓ in AD	[104]
Progranulin	Mut AD* n* = 130 NC *n* = 85	CSF	Progranulin ↑ in Mut AD	[108]
Progranulin	Cohort UCSF-MAC AD *n* = 186 MCI *n* = 118 NC *n* = 204 Cohort AddNeuroMedd AD *n* = 40 MCI *n* = 66 NC *n* = 95 Cohort ADNI AD *n* = 40 MCI *n* = 159 NC *n* = 240	Plasma CSF	No change in plasma progranulin Progranulin ↓ in CSF of AD patients	[109]
YKL-40	*n* = 318 cognitively healthy people at risk of Alzheimer's disease	Plasma	The concentration of YKL-_40_ ↑ with age, there was a negative association with the deposition of Aβ in the brain	[112]
sVCAM-1	AD *n* = 60 VaD *n* = 80 Non-dementia *n* = 40 NC *n* = 30	Plasma	sVCAM-1 ↑ in AD, vascular dementia and cerebrovascular disease without dementia (non-dementia)	[115]
sICAM-1	AD *n* = 25 NINDs *n* = 54 NC *n* = 15	Serum	sICAM-1 ↑ in AD and NINDs	[116]
IL-33 sST2	AD *n* = 30 MCI *n* = 30 NC *n* = 30	CSF Serum	IL-33 ↓ in CSF of AD and MCI patients IL-33 ↓ in serum of AD and MCI patients sST2 ↑ in serum of AD and MCI patients	[119]
EGF	Presymptomatic AD *n* = 259	Plasma	EGF ↓ in patients developing AD	[122]
EGF	AD *n* = 112 Parkinson's disease *n* = 236 MCI *n* = 396 NC *n* = 56	Plasma	EGF ↓ AD and MCI	[123]
Pancreatic polypeptide	AD *n* = 112 MCI *n* = 396 NC *n* = 58	Plasma	The level of CSF Aβ_42_ and the ratio of T-tau/Aβ_42_ correlate with the plasma level of the pancreatic polypeptide	[124]
HAGH* CASP8* EIF4EBP1* UNC5C* RGMB* JAM-B* TRAIL* SMOC* KYNU* sLDLR* tPA* *—only selected markers with the same change in CSF and plasma are listed for clarity	AD *n* = 176 MCI *n* = 131 NC *n* = 565	Plasma CSF	HAGH ↑ in AD, CSF and plasma CASP8 ↑ in AD, CSF and plasma EIF4EBP1 ↑ in AD, CSF and plasma UNC5C ↓ in AD, CSF and plasma RGMB ↓ in AD, CSF and plasma JAM-B ↓ in AD, CSF and plasma TRAIL ↓ in AD, CSF and plasma SMOC ↓ in AD, CSF and plasma KYNU ↓ in AD, CSF and plasma sLDLR ↓ in AD, CSF and plasma tPA ↓ in AD, CSF and plasma	[127]
LYN* CD69* EIF4G1* PLXNA4* SNAP29* FGF-5* MMP-3* KRT19* CSF-1* PAPPA* *—only the top 5 up and downregulated markers are listed for clarity	Discovery cohort: AD *n* = 106 NC *n* = 74 Validation cohort: AD *n* = 36 NC *n* = 61	Plasma	LYN ↑ in AD CD69 ↑ in AD EIF4G1 ↑ in AD PLXNA4 ↑ in AD SNAP29 ↑ in AD FGF-5 ↓ in AD MMP-3 ↓ in AD KRT19 ↓ in AD CSF-1 ↓ in AD PAPPA ↓ in AD	[128]

AD, Alzheimer's disease; AOC3, amine oxidase copper containing 3; CBS, corticobasal syndrome; CD8A, cluster of differentiation 8A; CD164, cluster of differentiation 164, CETN2, centrin 2; CHIT1, chitinase 1; DLB, dementia with Lewy bodies; dvppa, semantic variant PPA; EGF, epidermal growth factor; FTD, frontotemporal dementia; GAMT, guanidinoacetate N-methyltransferase; GSAP, gamma-secretase activating protein; hK14, human kallikrein 14; ICAM-1, intercellular adhesion molecule; JAM-B, junctional adhesion molecule B; KLK4, kallikrein-related peptidase 4; LIF-R, the leukemia inhibitory factor receptor; LYN, tyrosine-protein kinase Lyn; MMP-10, matrix metalloproteinase 10; MSA, multiple system atrophy; NFKBIE, NFKB inhibitor epsilon; nfvppa,non-fluent variant primary progressive aphasia; Ng, neurogranin; PCA, posterior cortical atrophy PRKCQ; PDD, Parkinson's disease dementia; RGMB, repulsive guidance molecule BMP co-receptor b; PRDX1, peroxiredoxin 1; PSP, progressive supranuclear palsy; protein kinase C theta; SCD, subjective cognitive decline; SMOC2, SPARC-related modular calcium-binding protein 2; sST2, soluble interleukin 1 receptor-like 1; Thy-1 or CD90, cluster of Differentiation 90; TMSB10, thymosin beta 10; TRAIL, tumor necrosis factor ligand superfamily member 10; TRANCE, tumor necrosis factor related activation-induced cytokine; UNC5C, Unc-5 netrin receptor C; VaD, vascular dementia; VCAM-1, vascular cell adhesion protein 1; VPS37A, vacuolar protein sorting 37 homolog A; YKL-40 or CHI3L1; chitinase-3-like protein 1

## MicroRNA (miRNA) biomarkers of AD

Mature miRNAs are single-stranded RNA molecules of 20–25 nucleotides [134] that can regulate gene expression post-transcriptionally by binding to the 3'-untranslated region (3’UTR) of mRNAs and blocking protein synthesis or leading to the degradation of target mRNAs [135]. While the biological effect of miRNAs is achieved intracellularly, miRNAs are often found in extracellular space as a consequence of leakage from damaged cells (due to injury, chronic inflammation, apoptosis, or necrosis), active secretion via microvesicles (microparticles, exosomes or apoptotic bodies) and active secretion of protein-miRNA complexes (high-density lipoproteins, Ago2) [136, 137]. Encapsulation of miRNAs as well as their binding to proteins increases their stability in body fluids and offers possibilities to specifically analyze exosome or protein-bound fractions as biomarkers [138, 139].

**Fig. 2:**
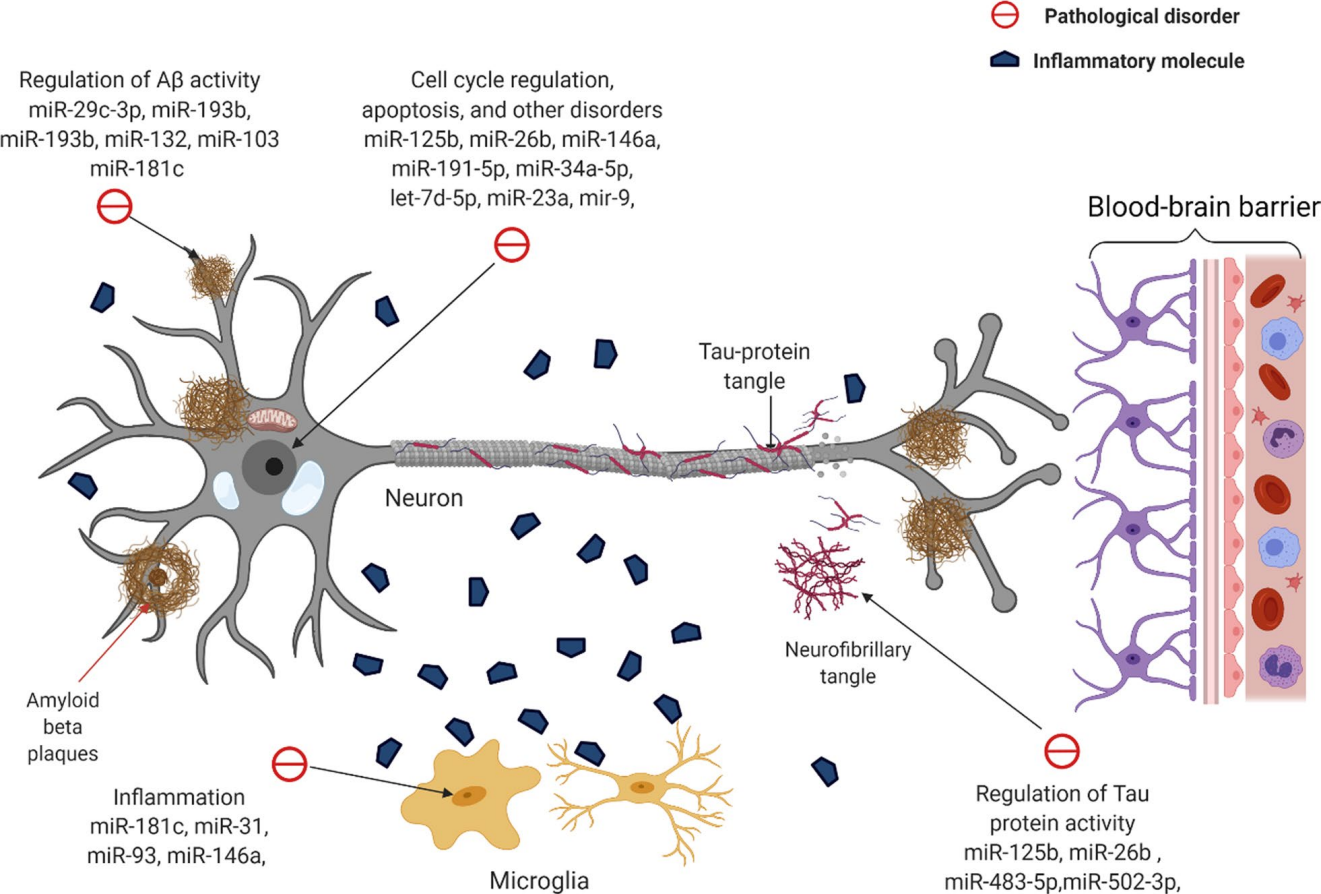
The effect of miRNAs on the pathology of AD. This image visualizes the effect of dysregulated miRNAs on neuronal degeneration. MiRNAs have an effect on the disruption of the cell cycle, regulation of Aβ metabolism, regulation of Tau-protein metabolism, and neuroinflammation

The levels of several miRNAs and their corresponding target mRNAs are changed under pathology and in the course of AD, affecting processes critical to the disease development and progression such as APP production, Tau phosphorylation and Aβ production (Fig. 2) [140]. Since miRNAs show a highly dynamic profile in AD, efforts have been made to characterize differentially expressed circulating miRNAs in body fluids of AD patients, such as CSF, plasma and serum [141]. The involvement of miRNAs in the pathogenesis of AD, the ability to detect them in easily accessible body fluids and the relatively high stability of miRNAs compared to mRNA make them an attractive target for AD biomarker discovery [42, 141, 142]. Among possible sources of miRNA, exosomes have received the highest interest, due to their unique features such as containing high concentrations of miRNA (3- to 4- fold higher than in serum) and mirroring pathological states due to the non-random packaging mechanisms [143]. In addition, exosomes have the potential to cross the BBB and circulate in bioliquids, allowing for relatively non-invasive, indirect detection of CNS pathologies in blood [144].

There are several convenient and reliable methods to screen for miRNAs. One leading method is the miRNA-adapted quantitative reverse-transcription polymerase chain reaction (RT-qPCR). Due to the small size of miRNA, cDNA synthesis is aided *inter alia* by stem-loop RT primers or polyadenylation followed by anchored dT primer hybridization and cDNA synthesis [145]. These and other approaches are commonly accessible through miRNA RT-qPCR kits, making it the first choice when the exact sequence of a target miRNA is known and when relatively low numbers of targets are analyzed. For bulk analysis, microarray hybridization and NanoString sequencing are convenient miRNA quantification methods [146]. The most powerful tools are the next-generation sequencing (NGS) methods which are particularly useful in the discovery phase, when identification of novel or rare miRNAs is desired in addition to their quantification. However, it is important to note that the NGS methods for miRNA quantification are burdened with sequence-related bias, require robust bioinformatic pipelines for reliable analysis and are usually more expensive than the aforementioned methods [147]. Therefore, methods are used according to the needs and the budget of a laboratory and often a combination of sequencing or microarray methods (bulk analysis and discovery phase) is used together with RT-qPCR (validation and cohort analysis) to find differentially expressed miRNAs.

MCI as a transitional state between normal aging and AD, is frequently targeted in AD biomarker discovery. RT-qPCR combined with differential correlation analysis was used in a study of 20 pairs of miRNAs as potential biomarkers of MCI in blood plasma, in a group of 76 patients (42 controls and 34 MCI) [148]. The analysis revealed that several pairs of miRNAs, namely, miR-191-miR-101, miR-191-miR-125b and miR-103-miR-222, have the highest potential to distinguish MCI from controls. The results of the study are corroborated by independent findings indicating that miR-191, miR-103, miR-125b and miR-222 are dysregulated in blood samples of AD patients and could serve as biomarkers of the disease [149–152]. The candidate biomarkers for investigation are frequently selected from miRNAs with known roles in AD development, such as miR-193b which regulates *APP* expression. miR-193b decrease has been shown in hippocampus, CSF and blood serum of transgenic APP/PS1 mice as a model of AD. Subsequent analysis in 43 MCI, 51 AD patients and 7 healthy controls revealed that miR-193b is decreased in MCI patients compared to controls, and even more decreased in AD patients. Taken together, these results suggest that miR-193b could be a potential biomarker of early AD [153]. Other miRNAs such as miR-34a-5p and miR-545-3p can also serve as additional biomarkers of early AD. However, the existing variability of miRNA analysis across different hospitals is an obstacle to the clinical use of miRNA [154]. The authors also suggest that age is a potential factor affecting the results of miRNA analysis in different cohorts. Indeed, recent studies showed that age can strongly influence the miRNA profile in blood [155], therefore it is particularly important to use age-matched cohorts in miRNA biomarker discovery and validation. Xie et al. showed that miR-206 and miR-132 are significantly downregulated in the serum of MCI patients compared to the healthy, age- and gender-matched controls. The obtained results correlated with the Montreal cognitive score in patients with MCI, which indicates the potential of this pair of miRNAs as a biomarker for the diagnosis of MCI [156]. Other promising miRNA biomarkers of MCI and its progression are miR-146a and miR-181a. In a two-year study, an increased circulation of these miRNAs was detected in patients with MCI, who later converted to AD [157].

An important area of research on miRNA markers is the search for miRNAs that will allow differentiating between stages of AD. Using miRNA microarray analysis followed by verification with RT-qPCR, researchers examined 84 miRNAs in serum and CSF of seven patients with AD and six patients with NINDs. The results revealed a significant decrease in miR-125b, miR-23a, and miR-26b in the serum of AD patients. The results of this study were confirmed in a larger, independent cohort composed of 15 patients with AD, 12 with NINDs, 8 patients with inflammatory neurological diseases, and 10 patients with frontotemporal dementia. These findings indicate the possibility of using miR-125b and miR-26b as markers for differential diagnostics of AD [42]. In a large study involving 465 participants, RT-qPCR and machine learning models were used to select biomarker candidates from 21 circulating miRNAs in AD patients [158]. The initial analysis identified 11 miRNAs with significant alterations in expression between AD and healthy age-matched controls. Of those, miR-532-5p had the most significant correlation with AD. Among the miRNA panel, miR-26a and 26b-5p were significantly correlated with the Mini-Mental State Examination (MMSE) score, but they were significantly deregulated at the initial stage of AD [158]. miR-103 is also an miRNA significantly associated with AD and its potential as a biomarker of AD has recently been corroborated by an independent study in a cohort of 120 AD, 120 PD and 120 healthy participants. Results showed that miR-103 was reduced in patients with AD compared to the controls. Furthermore, miR-103 levels were positively correlated with the MMSE score and negatively correlated with the severity of dementia in patients with AD in a study that included a cohort of 120 patients with AD, 120 patients with PD who served as disease controls, and 120 healthy controls [151].

**Fig. 3:**
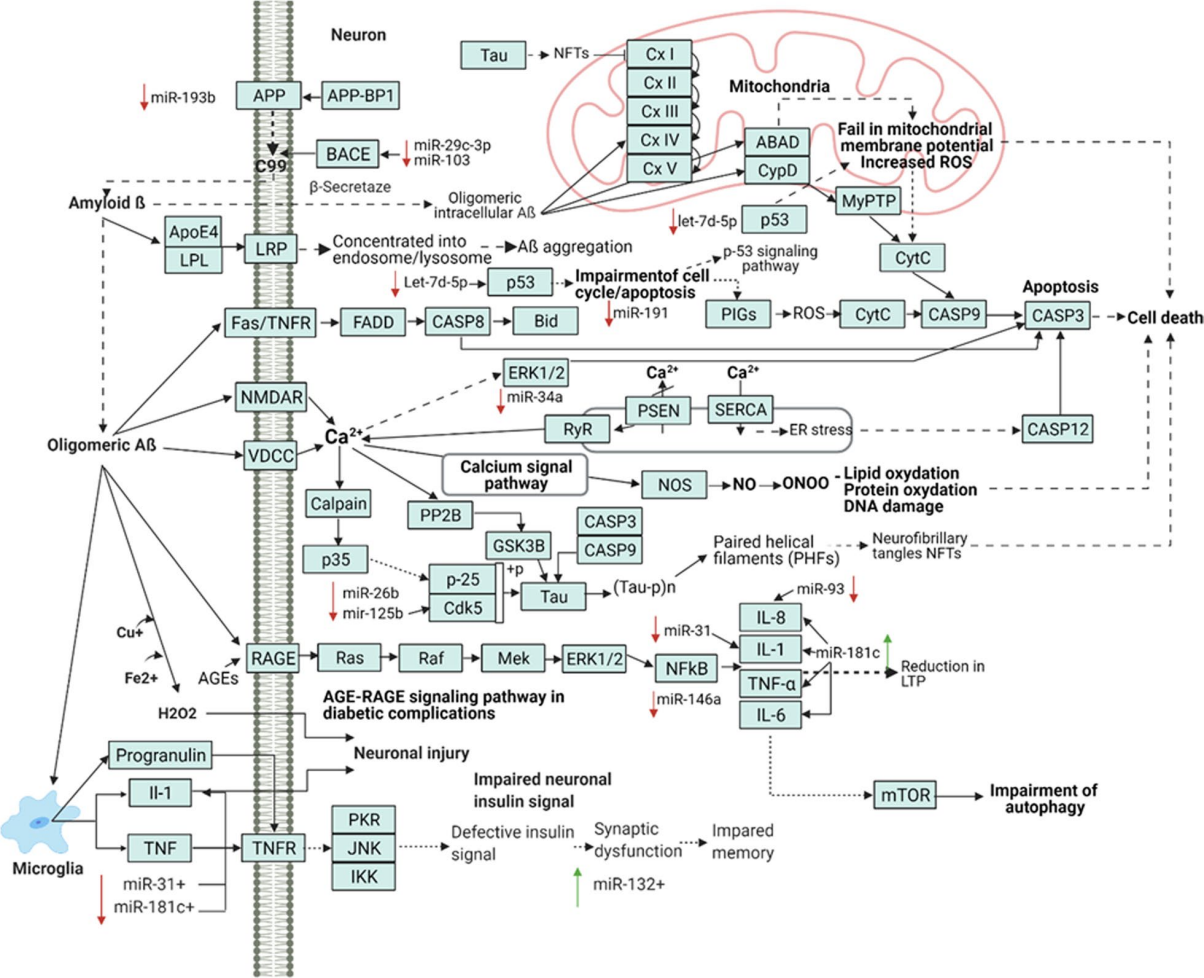
Association of AD pathways with miRNAs with potential for diagnostic applications. As one of the key pathological pathways of AD development, Aβ has effects on the development of mitochondrial dysfunction, oxidative stress, and induction of the calcium signaling pathway. The calcium signaling pathway affects the development of apoptosis via lipid oxidation, protein oxidation, and DNA damage, leading to cell death. One of the mechanisms of neuronal damage is represented by hyperphosphorylated Tau protein, which causes neurofibrillary degeneration. The image also shows the effect of inflammatory factors on neuronal damage. TNF, tumor necrosis factor; TNFR, tumor necrosis factor receptor; IKK, IκB kinase; PKR, protein kinase R; JNK, c-Jun N-terminal kinase; RAGE, receptor for advanced glycation endproducts; MEK, mitogen-activated protein kinase; ERK1/2, extracellular signal-regulated kinases; NF-κB, nuclear factor kappa-light-chain-enhancer of activated B cells; IL, interleukin; mTOR, mechanistic target of rapamycin; VDCC, voltage-dependent calcium channel; Cdk5, cyclin dependent kinase 5; PP2B, protein phosphatase-2B; GSK3B, glycogen synthase kinase 3 beta; NMDAR, N-methyl-D-aspartate receptor; NOS, nitric oxide synthase; RyR, ryanodine receptors; PSEN, presenilin; SERCA, sarco/endoplasmic reticulum Ca2+-ATPase; FADD, Fas-associated protein with death domain; BID, BH3 interacting-domain death agonist; CytC, cytochrome complex; APP, amyloid precursor protein; APP-BP1, amyloid precursor protein-binding protein 1; BACE1, beta-site APP cleaving enzyme 1; Cx proteins I-V, electron transport chain enzymes (complexes I-IV) and the ATP synthase (complex V); ABAD, amyloid beta-binding alcohol dehydrogenase; CypD, mitochondrial peptidyl-prolyl *cis*–trans isomerase D

MiRNAs are involved in the development of AD, reflect its pathophysiology and respond to changes associated with the development of this disease. miRNAs can facilitate understanding of AD in terms of amyloid theory, Tau protein aggregation, neuroinflammation, oxidative stress, and cell cycle disorders (Fig. 3). For instance, miR-125b is upregulated in the AD brain and correlates with the increased expression of Tau kinase genes (*p53*/*CDK5R1*, *CDK5*, *ERK1* and *ERK2*) and decreased expression of Tau phosphatase genes *DUSP6* and *PPP1CA* [159]. It is noteworthy that *DUSP6* and *PP1CA* are validated targets of miR-125b and are regulated through miR-125b binding to the 3’UTRs of their mRNAs. The high level of miR-125b is also involved in the cell cycle through downregulation of the cyclin-dependent kinase inhibitor 2A, which leads to increased proliferation of glial cells [160]. MiR-125b is strongly upregulated by nuclear factor kappa-light-chain-enhancer of activated B cells, and it can contribute to the development of neuroinflammation by targeting complement factor-H (*CFH*) mRNA [161]. The above examples underscore the importance of miRNAs, showing that individual miRNAs such as miR-125b can have pleitropic effects in AD. The miRNAs with an experimentally validated role in AD development as well as their potential use as biomarkers are summarized in Table 2.

**Table 2:** miRNAs associated with Alzheimer’s disease

miRNA	Sample	Project conclusions	References
miR-125b	Serum, blood, CSF, blood plasma	Level of miR-125b is decreased in the serum of AD group compared to the control group. MiR-125b is upregulated in the AD brain, where it leads to the increased cyclin-dependent kinase 5 expression and tau hyperphosphorylation. MiR-125b downregulates the cell cycle inhibitor CDKN2, and increases proliferation of glial cells.	[42, 150, 159, 160]
miR-181c	Serum	Level of miR-181c is decreased in the blood of AD and MCI group compared to control group. MiR-181 participates in the fine-tuning of inflammatory processes in astrocytes, decreasing the production of TNF-α, IL-6, IL-1β and IL-8.	[150, 162, 163]
miR-26b	Serum, blood, CSF	Expression of miR-26b is downregulated in the serum compared to non-inflammatory neurological controls. MiR-26b induces proliferation of postmitotic neurons via targeting Rb tumor suppressor mRNA, which leads to activation of CDK5 kinase involved in Tau phosphorylation and apoptotic neuron death.	[158, 164]
miR-31	Serum	Level of miR-31 is decreased in the serum of AD group compared to control group. MiR-31 is downregulated in the brains of AD patients and AD mice. Overexpression of miR-31 reduces amyloid β in hippocampus of transgenic mice through direct targeting of *APP* and *BACE1* mRNAs.	[165, 166]
miR-146a	Serum	Level of miR-146a is decreased in the serum of AD group compared to control group. MiR-146a is connected to neuroinflammation, and is upregulated by NF-κB, a pro-inflammatory transcription factor. MiR-146a inhibits *LRP2* mRNA translation, which also leads to cell apoptosis.	[161, 165, 167]
miR-29c-3p	Serum	Level of miR-29c-3p is decreased in the serum of AD group compared to control group. MiR-29b-3p targets the *BACE1* mRNA. BACE1, also known as beta-secretase 1, promotes the formation of Aβ-plaques by producing Aβ peptides.	[168–170]
miR-19b-3p	Serum	Level of miR-19b-3p is decreased in the serum of AD group compared to the control group. MiR-19 inhibits the aluminum-induced apoptosis of neurons.	[168, 171]
miR-34a-5p	Blood plasma	Expression of miR-34a-5p is downregulated in the serum of AD group compared to control group. The expression of miR-34a is downregulated in response to Aβ, which leads to increased level of its target cyclin-D1 and cell cycle-related apoptosis.	[154, 172, 173]
miR-206	Serum	Level of miR-206 is increased in the serum of the MCI group compared to the control group. MiR-206 promotes cognitive decline by suppressing BDNF expression in the brain.	[156, 174]
miR-132	Serum	Level of miR-132 is increased in the serum of the MCI group compared to the control group. MiR-132 expression reduces the expression of nitric oxide synthase and oxidative stress in brain tissues via the p38 signaling pathway in a rat AD model.	[156, 175, 176]
miR-34c	Blood	Level of miR-34c is increased in the blood of AD and MCI groups compared to the control group. Increased miR-34c expression in hippocampal neurons in AD negatively regulates the density of the hippocampal dendritic spine.	[177–179]
miR-15b-5p	Blood plasma	Level of miR-15b-5p is decreased in the blood plasma of AD group compared to the control group. MiR-15b-5p targets the amyloid precursor protein mRNA and has a neuroprotective effect.	[180, 181]
miR-222	Serum	Expression of miR-222 is decreased in serum in the mild and moderate AD patients compared to the control group. Reduced expression of miR-222 in AD may contribute to cell cycle disruption by altering the expression of cyclin-dependent kinase inhibitor 1B.	[148, 182, 183]
miR-103	Blood plasma	Expression of miR-103 is decreased in the blood plasma of AD patients.	[148, 151, 158, 184]
miR-107	Blood plasma	Expression of miR-107 is decreased in blood plasma of AD and PD patients compared to the control group. MiR-107 targets the 3’-UTR of *BACE1* mRNA. Decreased expression of miR-107 increases the BACE1 protein level, which is responsible for the formation of toxic forms of Aβ.	[151, 158, 164, 185]

APP, amyloid precursor protein; BACE1, beta-site amyloid precursor protein cleaving enzyme 1; BDNF, brain-derived neurotrophic factor; CDK5, cyclin-dependent kinase 5; CSF, cerebrospinal fluid; MCI, mild cognitive impairment; NF-κB, nuclear factor kappa-light-chain-enhancer of activated B cells; TNF, tumor necrosis factor

## Ocular biomarkers of AD

The retina of the eye is a promising diagnostic target for AD. The eyes and the brain share a common embryological origin, having a similar cellular fate specification of embryologically related tissues. The anterior neural tube forms the eyes and then gives rise to the forebrain. Aniridia type II protein PAX6, which plays a key role in neurogenesis, is also key to the development of the visual field. The retina is part of the central nervous system and shares structural and functional features with the brain, including the presence of neurons, glial cells, distinct blood barriers, and strict regulation of endothelial cell proliferation [186–189]. Since the retina and the brain have similar pathogenic pathways, a link between AD and eye pathology has been established. This has been shown in AD patients with an impaired visual function including reduced corrected visual acuity, decreased visual contrast, and impaired eye mobility [190]. Interestingly, the optic nerve axons connecting the brain to the retina can facilitate the transport of beta-amyloid precursor protein synthesized in retinal ganglion cells (RGCs) to small transport vesicles [191]. Currently, research efforts are focused on detecting AD at the preclinical stage. The accumulation of Aβ in the retina in the early stages of AD and amyloid-related neurodegeneration [53, 192], as well as the correlation of retinal thickness at the early onset of AD with atrophy of the parietal cortex [193], suggests that AD is not only a cerebral but also an eye disease. The retina is the only extra-cranial extension of the central nervous system and could reflect pathological changes in the brain in neurodegenerative diseases. The use of retina as a diagnostic tool opens up avenues for diagnosis of AD at its early stages and helps to monitor the effect of AD therapy directed against Aβ aggregates.

In recent years, several studies have shown a correlation between AD and degenerative changes in the retinal layers. For example, thinning of ganglion cell and internal plexiform layers (GCIPL) has been observed in several studies [194–202]. OCT is currently an important diagnostic tool in ophthalmology that allows visualizing the transverse structure of the retina with micron resolution and measuring the retinal nerve fiber layer (RNFL). At the same time, this imaging technique allows in vivo non-invasive studies of the anterior structures of the eye and the retina, as well as changes in blood flow and blood oxygen saturation in the retina [203–205] with highest resolution compared to other non-invasive imaging methods [206]. Using OCT, it has been shown that patients with AD have significant thinning of the RNFL [200, 202, 207, 208]. This method can measure the reduction in retinal layer thickness or the characteristics of retinal blood vessels using fundus images between AD patients and healthy control groups [209]. In one study, a group of 24 MCI patients, 30 patients with confirmed AD, and 24 cognitively normal age-matched control subjects underwent OCT to measure the thickness of the RNFL. The results showed a noticeable reduction in RNFL thickness, especially in the inferior quadrant, in AD and MCI groups. Interestingly, AD patients also showed significantly thinner RNFL in the superior quadrant compared to controls [210]. The heterogeneous decrease in RNFL could be explained by the fact that the thicker nerve trunks undergo more substantial degeneration than the thinner sections in other areas.

In a two-year study, retinal changes in AD were examined with ultra-wide-band retinal imaging. Drusus deposits were found on the periphery of the retina in patients with AD but not in controls. In addition, AD patients showed a tendency to increase the number of druses and areas affected by these deposits compared to the controls. The authors also measured several retinal vascular parameters during the study and found a significant increase in the venular width gradient in AD patients, indicating more profound thinning of the vessels towards the periphery in the disease group. This thinning might indicate that the peripheral retina receives less blood supply and nutrients, thus giving insights into retinal degradation during AD [211]. These vascular parameters might be promising biomarkers of AD; however, it is necessary to validate the findings in a longitudinal study in a large cohort.

The lower peripapillary retinal nerve fiber layer (pRNFL) sector undergoes the greatest changes in AD, and this region has been proposed to be the most sensitive in detecting a cognitive decline attributed to the disease [210]. Among several distinct changes, amyloid aggregation parameters combined with retinal ganglion cell degeneration in the upper quadrant of the inner retinal layers, NFL, and ganglion cell layer (GCL) can distinguish the AD-specific ocular pathology from that in other neurodegenerative diseases, such as macular degeneration (AMD) and glaucoma [212–214]. Furthermore, studies have confirmed that Aβ aggregation combined with RGC degeneration in the upper quadrant of the inner retinal layers (NFL and GCL) could be used to distinguish AD-specific pathological changes in the retina from changes related with other diseases.

Despite a noticeable correlation between neurodegeneration in AD and degeneration in the retina, the use of ocular biomarkers is hindered by the fact that degenerative changes in the retina occur in many other diseases. For example, AD and age-related AMD share pathological signaling defects and disease mechanisms at the molecular and genetic levels [215], which can significantly complicate the use of retinal imaging for the diagnosis of AD.

## Vascular network of the retina

In addition to measuring degenerative processes in the retina, visualization of the macular choroid can serve as another ocular biomarker of AD. The vascular systems of the brain and the eyes have a number of structural and functional similarities, and the connection between AD and the retinal vascular system has been confirmed in several studies [216–219]. Researchers have observed reduced blood flow due to reduced retinal vein diameter, sparse and more sinuous retinal vessels, and reduced arteriolar and venular fractal chambers [52, 220] in patients with AD. A difference in the thickness of the choroid is also observed in people with AD compared to healthy controls. Spectral-domain OCT has shown that patients with AD have statistically significant thinning of the choroid, thus highlighting the importance of vascular factors in AD pathogenesis [221].

Patients with AD show a tendency to have thinning of the choroid and these changes deviate from those observed in the age-matched controls [221]. Measurement of the choroid may have important additional significance in the diagnosis of AD, but for the use of this area of the eye for the diagnosis of AD in the preclinical stage, additional clinical studies are needed. It should be noted, however, that several studies have failed to find statistically significant differences in degenerative changes in the retina between AD patients and healthy subjects [222, 223]. For example, in a study of 160 monozygotic twins (aged ≥ 60 years), there were no differences in the thickness of the retinal layer in the macula or pRNFL between Aβ+ and Aβ− individuals. A positive association between non‐displaceable binding potential (BP_ND_, a continuous measure for Aβ) and macular total retinal thickness was found in the inner ring, but it was not statistically significant after adjustment for multiple comparisons [209]. Such results may indicate that the diagnostic effectiveness of retinal thickness as a biomarker of AD is limited. These results can be partially explained by the fact that observable differences in the retina resulting from neurodegeneration could only be detected at a relatively late-stage of AD [224]. It could also be explained by the fact that the study measured the thickness of the retinal layer, rather than its volume. In another study in 48 AD patients and 38 NC subjects, retinal parameters, choroid thickness, macular vessel density, and foveal avascular area size were measured using three imaging techniques (fundus photography, advanced depth imaging OCT, and OCT angiography [OCTA]). However, results did not reveal any effect of the disease on retinal vascular parameters after adjusting for confounder effects [225]. An interesting longitudinal study has been undertaken to investigate the association between degenerative changes in RNFL and the CSF Aβ42/Tau ratio. The study involved two cohorts of cognitively healthy individuals divided based on the Aβ42/Tau CSF ratio (normal and AD prone group), and found no group differences in the macula or GCIPL, but a 10-μm difference in the thickness of the RNFL between the two groups [226].

In a prospective study, the densities of the radial peripapillary network (RPC) and RNFL were measured using the OCTA method. The study examined eyes of 29 patients with amyloid-dependent cognitive impairment associated with AD, 25 patients with subcortical vascular cognitive impairment, and 15 amyloid-dependent cognitively normal subjects. The results showed no correlation between RNFL degeneration and brain degeneration in patients with AD [227]. Such controversial results significantly diminish the chances of RNFL clinical translation as a biomarker of AD.

## Limitations and challenges

Several common challenges can be elucidated for all groups of proposed biomarkers. The common problems identified in previous studies include poor reproducibility of data, small patient populations, variability in study design, patient heterogeneity in age, sex and clinical stages of AD, inconsistencies in data processing, normalization and statistical analysis, short study durations, and a lack of validation of the results. To address these challenges, several measures could be taken to facilitate the development of clinically valid biomarkers. Larger participant enrollment would be beneficial for increasing the study power as many previous studies were conducted with less than 100 patients. However, several new studies such as the BioFinder include very large cohorts of well-characterized patients [74]. Many neurodegenerative conditions clinically overlap, and thus careful selection of investigated subjects is of paramount importance. Leveraging multiple methods of imaging aids could facilitate an increased number of data points, including measuring brain atrophy, hippocampal volume, and markers of brain hypometabolism. An important aspect in the development of new diagnostic methods is the standardization of sampling, analysis, and operating procedures. Application of computational algorithms for analysis of clinical data, such as degenerative changes in the retina or biomarker panels, might ensure reproducibility of the studies. This could also reduce the inter-hospital/laboratory variations during biomarker discovery, which is especially renowned for CSF biomarkers [228].

The specific types of biomarkers discussed also experience unique challenges. So far, the development of ocular markers of AD is hampered by the use of different generations of OCT technologies that results in potential differences in measurements. Also, the analysis of the retina in AD is difficult since degenerative changes in the retina are characteristic not only of AD but also of other neurodegenerative diseases and chronic diseases associated with aging, such as age-related macular degeneration, diabetes, and hypertension. Hence, researchers may experience difficulties when analyzing results due to overlapping clinical metrics.

The use of blood-based biomarkers, in turn, is limited due to an insufficient number of studies carried out in general populations or primary clinics since most of the studies are currently carried out in dementia clinics or specialized research centers. It is important to note that the real diagnostic potential of a biomarker of AD development should be evaluated in population-scale longitudinal studies. Evaluation in geriatric clinics is already biased by pre-selection of patients among whom the prevalence of AD is much higher than in the general population and the positive predictive values for a particular biomarker can thus be much lower than expected (i.e. yielding many false-negative results) [229, 230]. The key obstacle to the use of miRNAs for the diagnosis of AD is the high heterogeneity of the results, which does not allow them to be used clinically [41, 231]. This can be explained by the fact that miRNAs are associated with various physiological processes, are expressed in various brain regions, and affect different aspects of AD pathology. Additionally, many biological processes can be affected by multiple miRNAs simultaneously. For example, beta-site APP cleaving enzyme 1 is regulated by 10 different miRNAs [232]. Therefore, additional functional studies of miRNAs are important, and researchers need to identify the exact patterns of miRNA expression. These studies would clear the path to understanding the subtle mechanisms of miRNA regulation and their interconnection and impact on the pathogenesis of AD. Given the abundance of miRNAs, it seems feasible to identify a set or a panel of miRNAs that allows distinguishing different stages of AD from other types of dementia with high specificity.

## Conclusion

There has been a significant shift in the research for AD biomarkers due to rapid advancements in analytical and visualization techniques. Modern instruments and assays allow for more sensitive blood tests to detect the pathology. Despite the significant obstacles in the field, the route to overcome them seems to be manageable. The most robust strategy for biomarker (including miRNA) discovery in blood is to utilize a combination of biomarkers since such an approach could increase the accuracy and specificity of diagnosis [233, 234]. Longitudinal studies that look at combinations of several markers could be of great benefit. An important goal of these studies would be to set a diagnostic threshold that can be used to identify the initial stages of the disease.

One of the most promising avenues for biomarker application is the use as real-time indicators to track the effect of disease-modifying therapies for AD in clinical trials. Currently, there are more than 182 phase II and phase III clinical trials of AD therapy in the ClinicalTrials.gov database, in which the effects of therapies are typically monitored using CSF biomarkers and PET imaging. Some studies have started to adopt plasma biomarkers, such as Aβ_40_, Aβ_42_, P-Tau, and pro-inflammatory cytokines, as additional means to track AD progression (NCT03533257, NCT04228666, NCT04570644) [235–237], and we expect this trend to continue.

Overall, there is still a high need for biomarkers that will allow large-scale screening of patients in primary health care facilities to provide a reliable preliminary diagnosis of at-risk patients. They can subsequently be assessed using CSF markers and PET imaging for confirmation of the diagnosis. Blood-borne biomarkers could save significant funds on AD diagnostics compared to existing diagnostic methods, enabling low-cost diagnostic methods to a great number of people as primary screen. For instance, an approach that includes the combination of several miRNAs could be such a strategy. Specifically, miR-125b, miR-146a, miR-9, and miR-103, the most frequently investigated miRNAs, are the most promising diagnostic strategy for AD since they have demonstrated high sensitivity and specificity in studies.

Finally, there is a significant need for a deeper understanding of the relationship between the biomarker levels, lifestyle, and the pathophysiology of AD, including cognitive impairment. It is likely that the new promising AD biomarkers will help to accelerate the clinical development of effective therapeutic agents. Importantly, they may reduce the cost of the management of the disease and facilitate better designs of clinical trials.

## Data Availability

All information used herein was obtained from peer-reviewed publications or from publicly available data on clinical trials.

## Abbreviations

Aβ: Amyloid-β
AD: Alzheimer’s disease
ADNI: Alzheimer's Disease Neuroimaging Initiative
APP: Amyloid precursor protein
APOE: Apolipoprotein E
BBB: Blood–brain barrier
CSF: Cerebrospinal fluid
EGF: Epidermal growth factor
ELISA: Enzyme-linked immunosorbent assay
FDG: ^18^F-2-fluoro-2-deoxy-D-glucose
GCIPL: Ganglion cells and internal plexiform layers
GCL: Ganglion cell layer
ICAM-1: Intercellular adhesion molecule
IL: Interleukin
IP-MS: Immunoprecipitation-mass spectrometry
LBD: Lewy body dementia
MCI: Mild cognitive impairment
MMSE: Mini-Mental State Examination
MRI: Magnetic resonance imaging
NC: Normal cognition
NFL: Neurofilament light
NGS: Next-generation sequencing
OCT: Optical coherence tomography
OCTA: OCT angiography
PET: Positron emission tomography
pRNFL: Peripapillary retinal nerve fiber layer
P-Tau: Phosphorylated Tau
RGC: Retinal ganglion cells
RNFL: Retinal nerve fiber layer
RT-qPCR: Quantitative reverse transcription polymerase chain reaction
SCD: Subjective cognitive decline
SIMOA: Single molecule array
sST2: Soluble interleukin 1 receptor-like 1
T-Tau: Total-Tau
TREM2: Triggering receptor expressed on myeloid cells 2
VCAM-1: Vascular cell adhesion protein 1
